# Air Quality and Health

**DOI:** 10.3390/ijerph15112399

**Published:** 2018-10-29

**Authors:** Giorgio Buonanno, Otto Hänninen

**Affiliations:** 1 Department of Engineering, University of Naples “Parthenope”, 80121 Naples, Italy; 2 Department of Public Health Solutions, National Institute for Health and Welfare, FI-70701 Kuopio, Finland; otto.hanninen@thl.fi

In the Editorial “Air pollution and health” that appeared in Environ. Sci. Technol., 1970, 4 (2), pp. 87–87, Michael Bowen wrote: “*It is an unfortunate—but nevertheless accurate—characteristic of such industrialized societies as ours that the richest areas usually are the dirtiest. The old Yorkshire saying “Where there’s muck, there’s brass” puts it quite succinctly, if smugly. Even today, industrial managers in America sometimes smilingly refer to the acrid stench from their profitable plants as “the smell of money”*” [1].

50 years passed, but we are now witnesses that something has changed. We are no longer convinced that dirty air is an unavoidable adjunct to a booming economy, and there is evidence that air quality standards adopted can improve air quality in urban areas and workplaces. Industrial pollution does not dominate air quality due to improved emission controls and technology, while at the same time, traffic and residential combustion have increased their role. Thus, in Western countries, the choice between being poor but healthy or rich but unhealthy is overcome.

Nevertheless, environmental exposures are still associated with substantial mortality and burden of disease in the developed world (Landrigan et al., 2017 [2]), and even if we were able to significantly improve the quality of the ambient air, not so much has changed in indoor environments (mainly in our homes), where we spend more than 90% of our time. The main cause of this delay is the lack of awareness of the health risks present in indoor environments deriving from sources that, by custom, we consider “natural” and healthy. This is perhaps the biggest challenge that awaits us, and we hope that in 50 years even this editorial could be just a witness of past times.

This Special Issue brings together scientists from multiple disciplines to present their newest results concerning exposures in schools and nurseries, in and from traffic, perceived and observed related health effects, exposures to ultrafine particles, composition of exposures, and differences in toxicity of the exposures by emission sources. Several papers look at exposures in developing countries and most recent scientific epidemiological evidence collected by combining state-of-the-art exposures with observed health data. The way is paved forward, even if a lot of work needs to be done to establish safe and healthy living environments for mankind.
